# Lung Cancer Screening at US Hospitals for People Lacking Primary Care

**DOI:** 10.1001/jamanetworkopen.2024.42373

**Published:** 2024-10-31

**Authors:** William DeSantis, Oluwaseun Ayoade, Giorgio Caturegli, Daniel J. Boffa

**Affiliations:** 1 Yale School of Medicine, New Haven, Connecticut

## Abstract

This quality improvement study investigates how many US hospitals allow patients to schedule lung cancer screenings without a referral from a primary care practitioner.

## Introduction

Lung cancer screening ranks as one of the most effective cancer screenings available, saving one person’s life for every 320 screening computed tomography scans performed.^1^ However, less than 10% of the 15 million people who are eligible for lung cancer screening in the United States will participate, resulting in tens of thousands of preventable deaths each year.^2^

Cancer screening is typically arranged through primary care. However, roughly one-third of the US population lacks a primary care clinician,^3^ leaving millions to connect with lung cancer screening independently. Patients lacking primary care are advised to call hospitals directly for cancer screening.^4^ We explored the ability of hospitals to connect patients to lung cancer screening.

## Methods

In this quality improvement study, hospitals were telephoned regarding lung cancer screening via their main number (Figure). A directed sampling of 527 US hospitals was performed first (ie, ≥8 hospitals per state, Commission on Cancer [CoC] status balanced) followed by a random sample of 500 hospitals. Per the Common Rule, we determined that this study was institutional review board exempt, and we complied with survey best practices according to the AAPOR reporting guideline where appropriate. More details appear in eMethods in Supplement 1. Excel version 2407 (Microsoft Corp) was used to conduct analyses.

**Figure.**
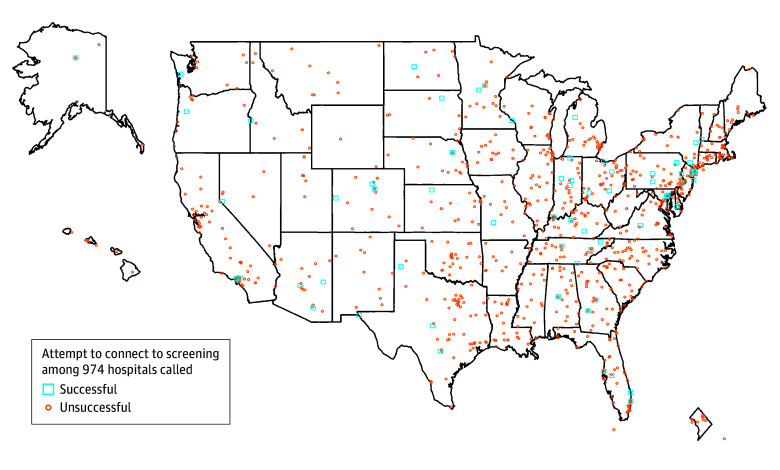
Map of Results of Phone Calls to Hospitals Investigators asked, “I want to get screened for lung cancer, could you help me?” A successful connection was considered if the call was routed to someone who could schedule an appointment with a clinician or other member of a formal screening program or if the call led to someone who could schedule the computed tomography scan (eMethods in Supplement 1). The random sample by chance included some hospitals in the directed sample, so the total number does not equal the random sample plus the directed sample.

## Results

Only 51 of the 527 hospitals (9.7%) initially contacted were able to connect to any component of a lung cancer screening process (Table).^5^ Calls failed most often because we lacked a primary care clinician’s order (317 calls [60.2%]). Similar results were seen in the random subset of 500 hospitals, irrespective of CoC accreditation status or listing on a screening locator website.

**Table.** Results of Screening Inquiries to Hospitals

Result of call	Hospitals, No. (%)			
	Directed sample cohort (n = 527)^a^	Random sample		
		Random sample cohort (n = 500)^b^	CoC accredited (n = 144)	Listed on ACR website (n = 125)^c^
Successful connection to screening process	51 (9.7)	19 (3.8)	10 (6.9)	9 (7.2)
Unsuccessful, needed to have a primary care practitioner	317 (60.1)	261 (52.2)	77 (53.5)	68 (54.4)
Unsuccessful, other reason^d^	159 (30.2)	220 (44)	57 (39.6)	48 (38.4)

Abbreviations: ACR, American College of Radiology; CoC, Commission on Cancer.

^a^ Directed sampling refers to sampling of US hospitals to achieve specific distribution endpoints (eg, ≥8 hospitals per state, roughly half CoC accredited) (eMethods in Supplement 1).

^b^ Random sample was taken completely at random (eMethods in Supplement 1).

^c^ Refers to lung cancer screening locator website from the American College of Radiology.^5^

^d^ Roughly half of the unsuccessful calls for other reasons were because of a phone tree that did not include an option for screening and did not offer an option to speak to a person or led to a voicemail, which we left but was not returned. The next most common reasons were being disconnected or being placed on hold for more than 10 minutes. Additional reasons include being told screening was not offered or the main hospital number was no longer a working number.

## Discussion

The need for primary care involvement could pose an important barrier to lung cancer screening, as 100 million people in the US currently lack access to a primary care practitioner.^3^ While primary care is not a specific requirement for lung cancer screening reimbursement, primary care practitioners can perform several of the required steps. Most notably is a shared decision-making visit, in which patients are assessed for eligibility for screening, counseled on the potential findings, and informed of the need to continue with yearly scans. This is critical as many patients will require additional workup for indeterminate pulmonary nodules (most of which are benign), and much of the benefit of screening is derived from the follow-up scans. Many screening programs include clinicians to serve this role for patients lacking primary care.

A far more challenging role to reassign from primary care is the need to follow incidental findings. Approximately 34% of screened patients will have a computed tomography scan finding unrelated to lung cancer that requires follow up (eg, adrenal nodule, aortic aneurysm).^6^ As a result of this follow-up requirement, walk-in or mobile screenings, which allow medically disenfranchised women to be screened for breast cancer, are currently less feasible for lung cancer. One approach would be to have selected patients self-manage steps of the evaluation of incidental findings, such as scheduling follow-up testing. This would be reserved for low-acuity findings, which represent by far the widest part of the funnel draining primary capacity. Our representation of individuals inquiring about screening could be a limitation, as it is possible that a more persistent or medically savvy caller may have been more successful.

There is no single solution to the puzzle of why lung cancer screening is so heartbreakingly underutilized. The primary care issue is clearly not the only answer, as there are likely millions of screen-eligible people with an established primary care practitioner who are not being screened. However, efforts to motivate the public to pursue lung cancer screening must be met with equal efforts to provide pathways for them to connect, particularly among the medically disenfranchised.

## Notes

Supplement 1.
eMethods.

Supplement 2.
Data Sharing Statement
