# Supplementary material for: Lung Cancer Screening at US Hospitals for People Lacking Primary Care
Source: JAMA Netw Open. 2024 Oct 31;7(10):e2442373. doi: 10.1001/jamanetworkopen.2024.42373 (PMC11528307; doi:10.1001/jamanetworkopen.2024.42373)
Supplement: Supplement 1. — eMethods. [file jamanetwopen-e2442373-s001.pdf]

# Supplemental Online Content

DeSantis W, Ayoade O, Caturegli G, Boffa DJ. Lung cancer screening at US hospitals for people lacking primary care. *JAMA Netw Open.* 2024;7(10):e2442373. doi:10.1001/jamanetworkopen.2024.42373

**eMethods.**

This supplemental material has been provided by the authors to give readers additional information about their work.

© 2024 DeSantis W et al. *JAMA Network Open.*

# eMethods.

This project began as a quality assurance effort to examine outlets for patients lacking primary care to connect with lung cancer screening. More specifically, much of the public facing messaging concludes with having people “ask your primary” https://www.latimes.com/paid-posts/?prx_t=rbsIABQhjA_EQQA&ntv_acpl=1066236&ntv_acsc=0&ntv_ot=0&ntv_ui=a5d9b7a1-2ac0-41b1-80b3-495c84283ab1&ntv_ht=PH_IZQA.
Recognizing that many people lack primary care, we investigated options for them to connect with screening. Individuals lacking primary are advised to call hospitals directly (see below supplemental posting from American Cancer Society), which in our opinion is completely reasonable. As a quality assurance project, we began calling hospitals focusing on Commission on Cancer accredited hospitals to evaluate the viability of this as an option to connect. We quickly identified a concerning signal and realized the need to upscale a more structured inquiry.

As a result, the hospital cohort was generated in two steps. The first 527 hospitals were identified using a directed sampling, with efforts to ensure specific representation into the dataset. More specifically, the directed sampling subset hospitals were chosen from publicly posted lists with no intentional inclusion efforts other than to include at minimum of 8 hospitals from every state, choose hospitals with outpatient services, and to represent an approximately even mix of Commission on Cancer (CoC) accredited hospitals and those that are not CoC accredited hospitals. More specifically, hospitals were identified using the American College of Surgeons (ACS) hospital/facility search tool, selecting for CoC-accredited cancer programs. Non-accredited hospitals were identified using the American Hospital Association facility search tool, and then checked with the ACS search tool to make sure they did not have CoC accreditation. At least 8 hospitals were chosen from each state. We attempted to minimize the number of hospitals affiliated with the same network.

A second cohort of 500 hospitals was generated completely at random by placing the full list of US hospitals list of all hospitals registered with Medicare: https://data.cms.gov/provider-data/dataset/xubh-q36u into a random number generator https://www.calculatorsoup.com/calculators/statistics/random-number-generator.php. Results from calls to these 500 hospitals were compared to the first cohort to potentially expose any selection bias in the original cohort. We examined the subset of hospitals that were accredited by the CoC in the random sample. We also examined the subset of hospitals that were included in the American College of Radiology screening locator https://www.acr.org/Clinical-Resources/Lung-Cancer-Screening-Resources/LCS-Locator-Tool.
The conversation between the hospital and study team was framed by a template, that began with the question “I want to get screened for lung cancer. Could you help me out with that?” If the hospital representative asked about insurance, we gave the answer of a private company (typically Blue Cross). If a phone tree was an option, we waited until the end, and asked for operator, imitating a person with limited familiarity with lung cancer screening or hospital call routing.

If we were asked to leave a phone number, we left a phone number of the research team member. If the hospital called back, they were coded appropriately. If no call back was received in two weeks, it was considered a failure to connect.
If placed on hold for more than 10 minutes, the call was terminated and counted as an unsuccessful connection (10 calls).

© 2024 DeSantis W et al. *JAMA Network Open.*

The vast majority of calls were between 9am and 4pm in the local time zone of the hospital. However, a few night and weekend calls were made (around 1%), as this is likely when some potentially eligible people would be able to call.

We wanted to minimize the time taken from the hospital phone teams, therefore we did not actually complete any scheduling interaction (i.e. give information for appointment to be scheduled). Therefore, it is possible that additional barriers exist downstream of our initial inquiry.

Outcomes of calls were coded as 1) Successful, if someone at the hospital began taking information to enroll us in the process, 2) Unsuccessful because of the need for a primary (referral or order for the CT scan), 3) Unsuccessful because of a "dead end" - most typically the hospital operator did not know who to connect us with and the call ended (29% of random subset), 4) Unsuccessful, because the facility did not offer lung cancer screening (6% of the random subset), 5) voicemail, where we were asked to leave a number, if we were called back, then we coded the call appropriately above, if no call back in 2 weeks, then the call was deemed a failure (9% of random subset). We recognize that some of the "dead end" calls could have been a reflection of the experience and knowledge of a specific operator. However, we wanted to mimic "real world" call experience, and it would be unlikely that a person would try to call a hospital again after a dead end.

The figure was created using STATA. A shapefile that can draw a USA map (http://pped.org/) was imported and then we added an overlying scatterplot of the hospital coordinates, obtained from an online search (https://www.gps-coordinates.net/).

© 2024 DeSantis W et al. *JAMA Network Open.*
