# Supplementary material for: Lung Cancer Screening at US Hospitals for People Lacking Primary Care
Source: JAMA Netw Open. 2024 Oct 31;7(10):e2442373. doi: 10.1001/jamanetworkopen.2024.42373 (PMC11528307; doi:10.1001/jamanetworkopen.2024.42373)
Supplement: Supplement 2. — Data Sharing Statement [file jamanetwopen-e2442373-s002.pdf]

Data Sharing Statement

DeSantis. Lung Cancer Screening at US Hospitals For People Lacking Primary Care. *JAMA Netw Open*. Published October 31, 2024. doi:10.1001/jamanetworkopen.2024.42373

Data
Data available: No

Additional Information
Explanation for why data not available: no patient information collected
